# Anterior Capsulectomy Through Humeral Fenestration in Arthroscopic Arthrolysis for Elbow Stiffness Is Safe and Effective

**DOI:** 10.1016/j.asmr.2024.101029

**Published:** 2024-10-15

**Authors:** Clémence Lemaître, Antoine Senioris, Fabrice Duparc

**Affiliations:** a Department of Orthopedic and Traumatology Surgery, Rouen University Hospital, Rouen, France; b Orthopedic Surgery, Clinique Mégival, Saint Aubin-sur-Scie, France; c Centre d’études et des transformations des activités physiques et sportives (CETAPS, EA 3832), Mont-Saint-Aignan, France

## Abstract

**Purpose:**

To evaluate arc of motion and complications following transhumeral anterior capsulectomy through a purely posterior approach with the Outerbridge-Kashiwagi procedure in treating elbow stiffness.

**Methods:**

Patients who were treated for elbow stiffness between April 2003 and February 2023 were retrospectively identified. The inclusion criteria were an extension/flexion arc deficit of at least 30° and treatment with arthroscopic arthrolysis through posterior and posterolateral portals with humeral fenestration. Elbow joint range of motion and the Mayo Elbow Performance Score were assessed preoperatively, intraoperatively, at 6 weeks, and at final follow-up. The follow-up ended when the elbow became asymptomatic again or when the recovery was considered stable. Postoperative complications were recorded.

**Results:**

A total of 30 patients (23 men/7 women; 31 elbows; 1 bilateral/29 unilateral) were included. Mean follow-up was 11.1 months (1-64). Mean joint amplitudes intraoperatively increased in all areas of mobility, including extension/flexion from 86° to 132.6° (*P* = .001) and pronation/supination from 163.9° to 179.7° (*P* = .025). At the longest follow-up, mean joint amplitude was increased from 86° to 118.9° (*P* = .002) in extension/flexion and from 136.9° to 173.9° (*P* = .022) in pronation/supination. The mean deficit was reduced from 54° to 21.1° (*P* = .001) in extension/flexion and from 16.1° to 6.1° (*P* = .006) in pronation/supination. The mean gain in the extension/flexion arc was 31.5° and 10° for the pronation/supination arc. Loss in flexion/extension was limited (mean: 14.2°, extreme: 50°). The study showed no neurologic complications.

**Conclusions:**

Arthroscopic arthrolysis of a stiff elbow using a purely posterior approach with anterior capsulectomy via the Outerbridge-Kashiwagi procedure was safe and effective. Clinical results showed improvement in joint range of motion in flexion/extension and pronation/supination, both intraoperatively and postoperatively, with no postoperative neurologic complications.

**Level of Evidence:**

Level IV, therapeutic case series.

After an elbow trauma, the most common complication is stiffness. Other nontraumatic causes of stiffness include degenerative osteoarthritis,^1^^,^^2^ inflammatory arthritis, hemophilic arthropathy, osteochondromatosis, prolonged immobilization, foreign bodies, and infectious causes. Joint arthrolysis must treat all these different lesions.^3^ The elbow joint amplitude required for daily life is at least 100° based on the study by Morrey et al.,^4^ which showed that 90° of everyday gestures could be performed with a functional arc of the elbow ranging from 30° to 130°. Loss of degrees in flexion appears to be more disabling than loss of the same number of degrees in extension. The therapeutic objective is to prevent stiffness. Once stiffness is present, the patient should receive the most appropriate treatment, ranging from medical therapy (analgesics, orthoses, rehabilitation) to surgery. If surgical management is indicated, conventional or arthroscopic arthrolysis may be proposed when articular cartilage is preserved.^5^^,^^6^ In the case of extensive joint damage, interposition arthroplasty or total elbow prosthesis should be considered.^7^^,^^8^

The basic arthrolysis procedures include anterior and posterior capsulectomy, resection of the olecranon and/or coronoid bone spurs, and removal of fibrous tissues in the olecranon and coronoid fossae. In the literature, open arthrolysis and arthroscopic arthrolysis, for comparable indications, lead to similar results.^9^^,^^10^ Kelberine and Kazal^9^ list studies reporting the results of either open or arthroscopic arthrolysis depending on the causes of stiffness, and the results are comparable:

- For post-traumatic causes, flexion/extension gain is between 35° and 45° open and arthroscopically.
- For arthritis causes, flexion/extension gain is around 35° open and arthroscopically.
- For mixed series, flexion/extension gain is between 25° and 40° open and arthroscopically.

Arthroscopic arthrolysis has become the treatment of choice.^11^, ^12^, ^13^, ^14^, ^15^ This technique offers advantages by reducing the morbidity of the procedure, in particular by avoiding the risk of adhesion and recurrence. Kodde et al.^16^ carried out a systematic review to summarize current literature and to compare gain in range of motion and the number of complications for open and arthroscopic arthrolysis. This study showed a higher complication rate in open arthrolysis (20% vs 5% in arthroscopy), with the main complication being reoperations for persistent stiffness.^16^

Arthroscopic arthrolysis is a difficult technique, requiring a high level of experience with arthroscopy. This procedure may present rare but serious complications,^17^ mainly neurologic due to the proximity of nerves (median and radial nerves). The article by Tsevan and Nicolay^17^ reported low overall complications of elbow arthroscopy (from 1.5% to 11%) and a low nerve injury rate (from 1.26% to 7.5%) depending on the study. The stage of anterior capsulectomy using an anterolateral arthroscopic approach is less commonly performed than open capsulectomy because accessibility is more limited with a high neurologic risk.^18^^,^^19^ Marois and Field^18^ highlight the need to pay close attention to the anterior inferior capsule, which should be approached with caution, as its violation puts branches of the radial nerve, specifically the posterior interosseous nerve, at risk.

Secondary loss of mobility in extension and flexion after extensive open arthrolysis raises the possibility of adhesion recurrence in sliding, as well as recurrence of fibrous tissues in the coronoid and olecranon fossae.^20^^,^^21^ Humeral fenestration (Outerbridge-Kashiwagi) was one of the procedures performed during open arthrolysis to avoid the risk of the recurrence of stiffness. Humeral fenestration for elbow arthrolysis is also known as the Outerbridge-Kashiwagi procedure.^22^ Initially described for the management of arthritic elbow stiffness,^23^ this method uses a posterior elbow approach to reach the anterior compartment via a humeral bone fenestration.^24^^,^^25^ It enables an anterior capsulectomy to be performed while avoiding neurologic risks and preventing secondary filling of the olecranon and coronoid fossae, which can lead to secondary loss of postrelease mobility. Given the reduced risk of neurologic complications, with equivalent results and a lower risk of recurrence, we decided to transpose this technique to our arthroscopic elbow surgery practice.^26^^,^^27^

The purpose of this study was to evaluate arc of motion and complications following transhumeral anterior capsulectomy through a purely posterior approach with the Outerbridge-Kashiwagi procedure in the treatment of elbow stiffness. Our hypothesis was that we could perform elbow arthrolysis by the arthroscopic transhumeral approach and avoid any neurologic complications.

## Methods

### Patients

**Fig 1:**
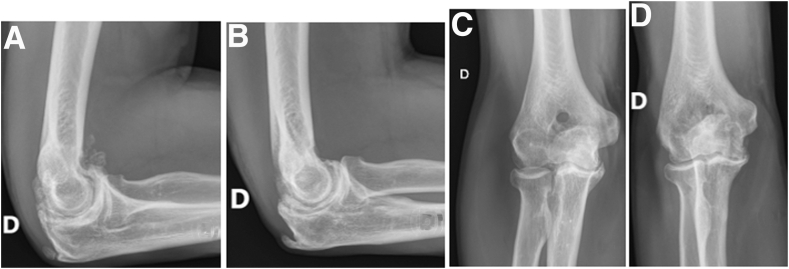
Right elbow radiographs. A lateral radiograph showing numerous osteochondromas preoperatively (A), explaining the elbow stiffness. Lateral (B) and anteroposterior (C & D) radiographs at 6 weeks and 1 year (D) respectively showed no recurrence of osteochondromas or fracture due to humeral fenestration.

Ethics committee approval was obtained for this study (number E2024-47). All patients gave informed consent for the use of their data. Patients who were treated for elbow stiffness using an arthroscopic method with humeral fenestration with the Outerbridge-Kashiwagi method between April 2003 and February 2023 were identified. The inclusion criteria were that the patient was over 18 years of age and had elbow stiffness with an extension/flexion arc deficit of at least 30° (compared with an average standard of 150°), with or without pronation/supination insufficiency, and associated or not with pain. The exclusion criteria were chronic instability of the elbow and removal of osteosynthesis material that would justify an open approach. Patients with missing data were not excluded. Each patient had anteroposterior and lateral conventional radiographs of the elbow before and after the operation to monitor the disappearance of osteochondromas and the evolution of the humeral fenestration (Fig 1). Some patients had a preoperative computed tomography scan or magnetic resonance imaging. No other complementary examination was systematic.

### Surgical Technique

Arthroscopic elbow arthrolysis via posterior and posterolateral approaches with humeral fenestration according to the Outerbridge-Kashiwagi method was performed for all patients.

**Fig 2:**
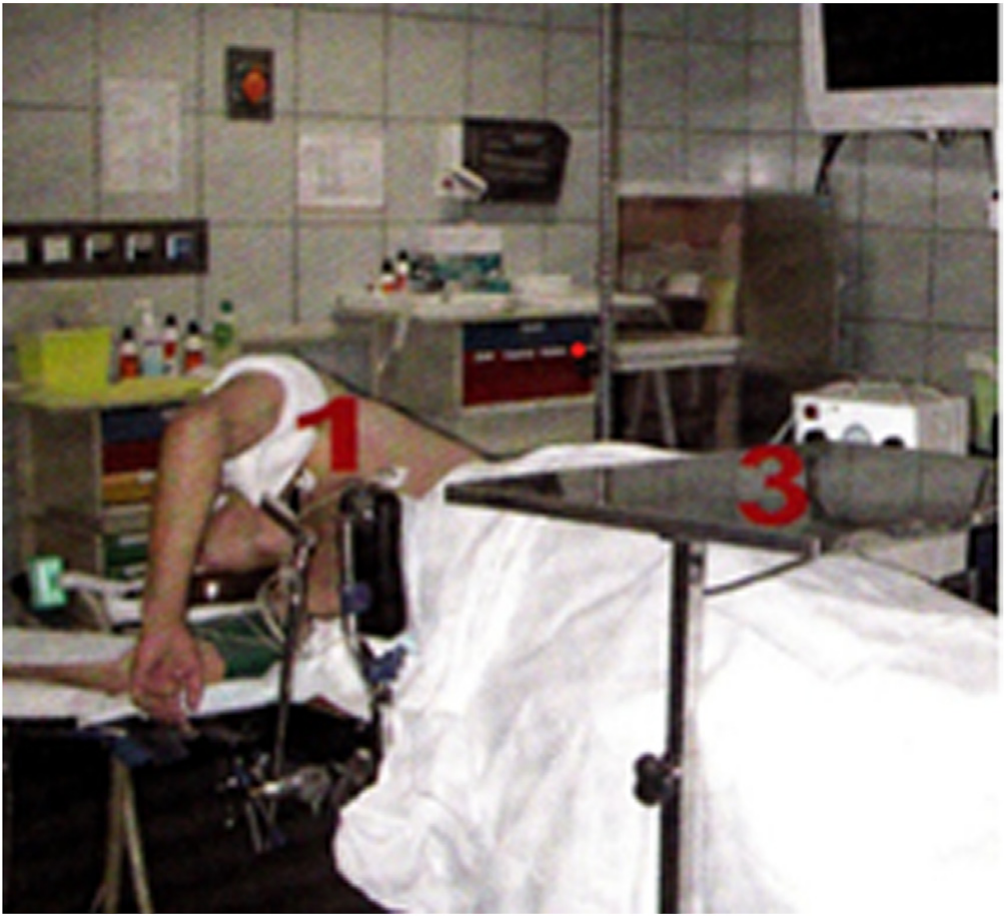
Patient installation. The patient is placed in a lateral decubitus position. The forearm is pronated, and the elbow flexed to 90° rests stably on an armrest. A tourniquet is placed at the root of the arm (1). The instruments are placed on a table above the patient (3). The arrows indicate the arthroscopic portals.

**Fig 3:**
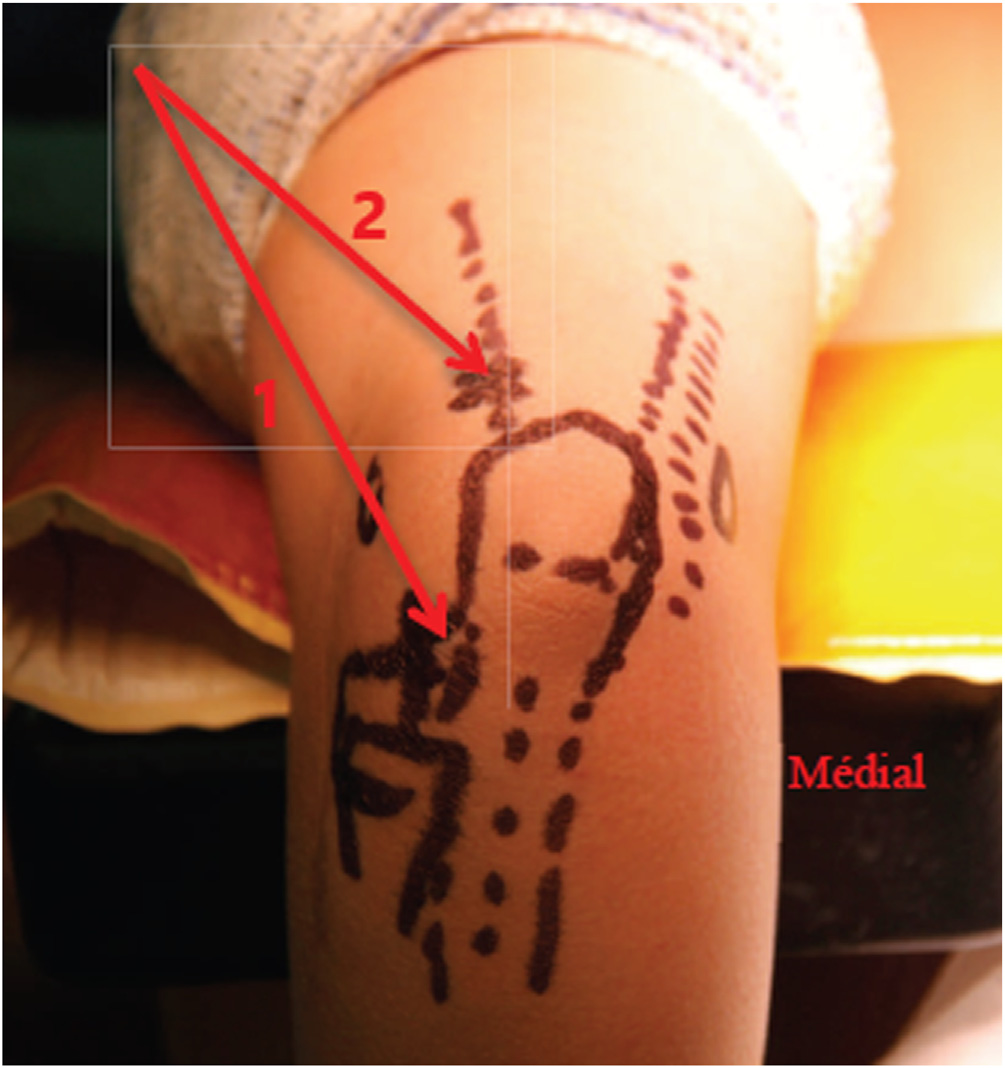
Surgical approaches. The arthroscopic surgery was performed via 2 approaches: a posterolateral approach, shown by the most distal arrow (1), to introduce the arthroscope and a posterior approach for other instruments, shown by the most proximal arrow (2).

**Fig 4:**
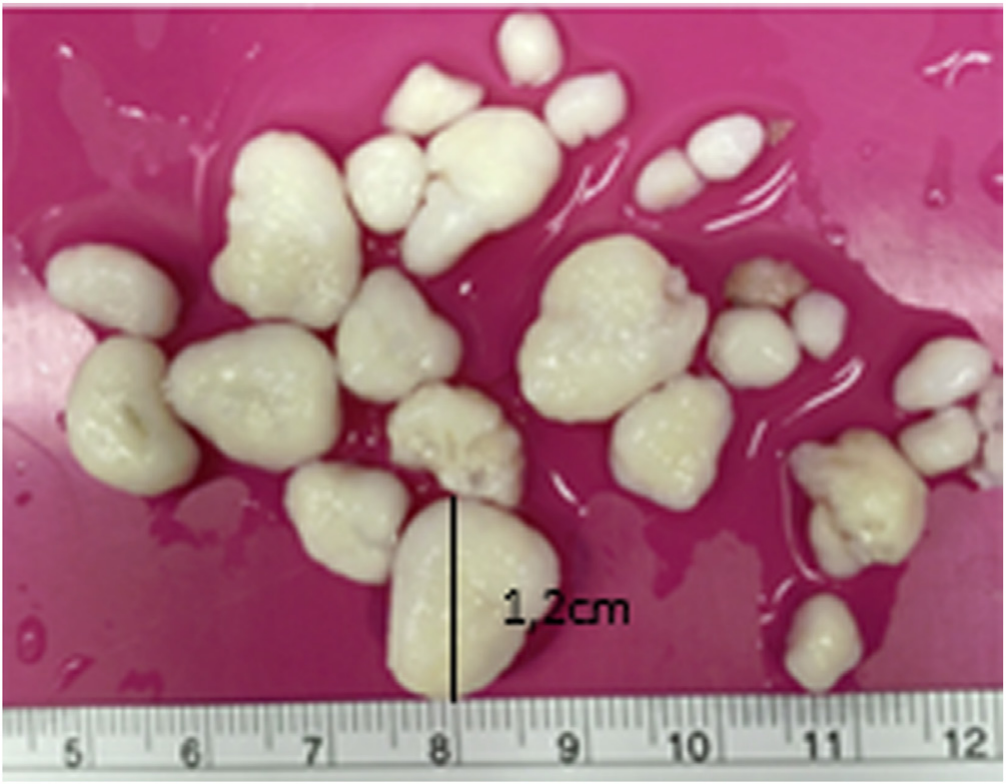
Osteochondromatous fragments. Postoperative photograph of a large number of osteochondromatous fragments removed with forceps using arthroscopy. The largest fragments measure over 1.2 cm.

**Fig 5:**
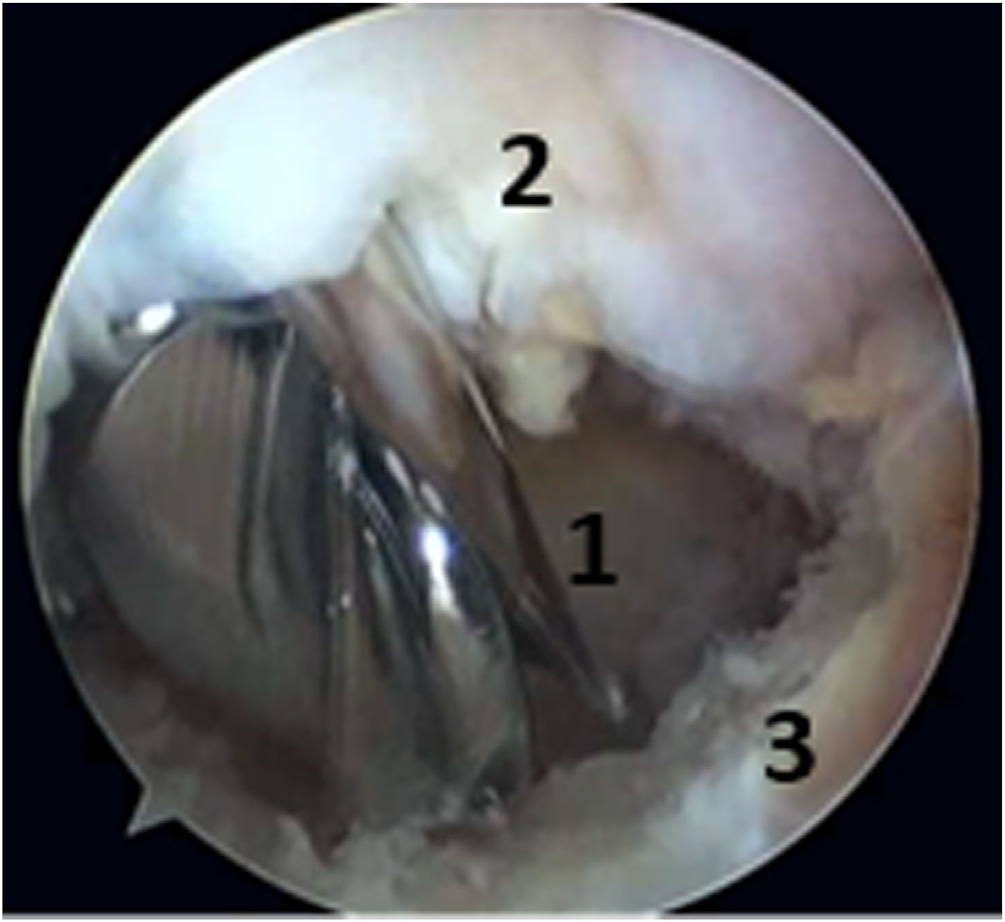
Arthroscopic joint cleanse. A burr (acromionizer) (1) was introduced through the posterior approach to the joint to release the fibrous tissue (2), the osteophytes, and the olecranon bone spur (3).

**Fig 6:**
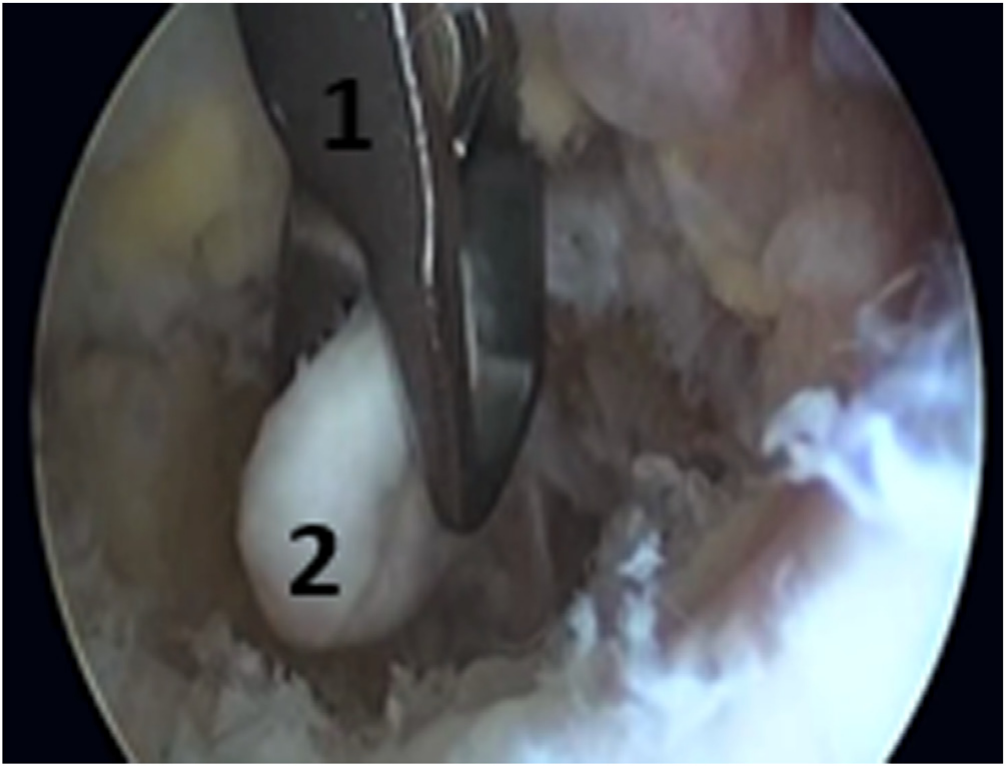
Extraction of foreign bodies. The extraction of foreign bodies (2) is realized with forceps (1) from the anterior compartment through the humeral fenestration.

**Fig 7:**
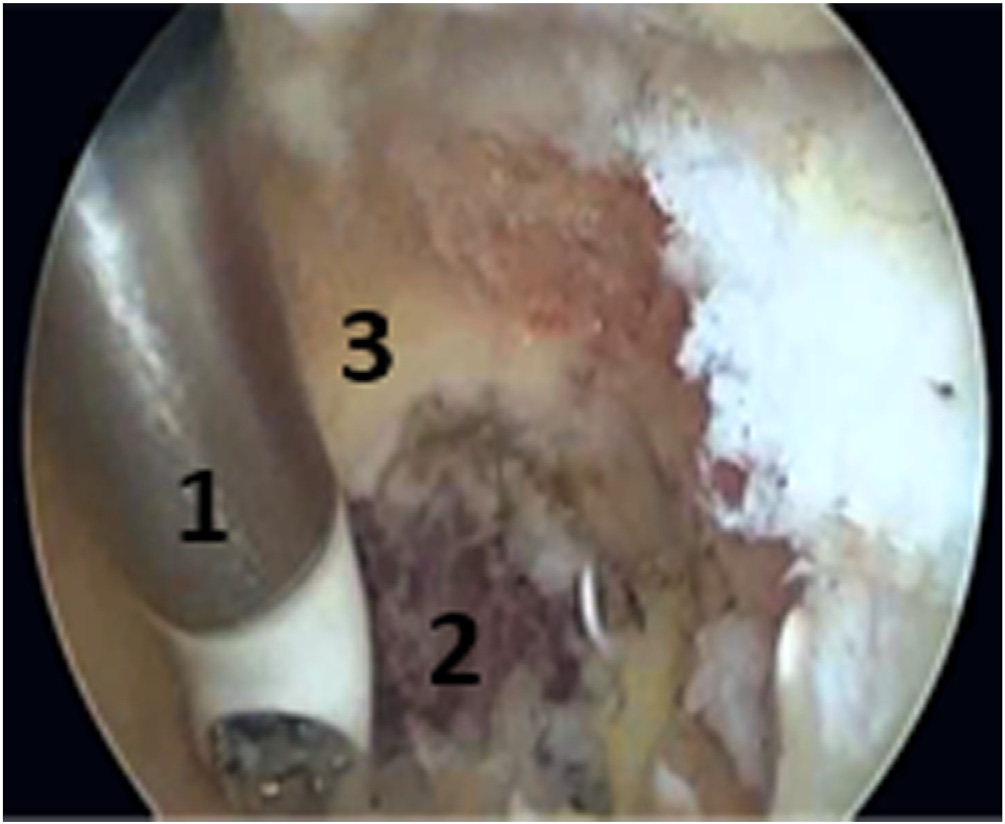
Anterior capsulectomy through the humeral fenestration. The anterior capsulectomy by radiofrequency energy (1) was performed up to the brachial muscle fibers (2) in front, followed by anterior synovectomy around the bone window (3) and then extended throughout the entire arthroscopic work area.

The patient underwent the operation under general anesthesia in the lateral decubitus position (Fig 2). A tourniquet was placed at the root of the arm. The arthroscopic surgery was performed via 2 approaches: a posterior portal and a posterolateral portal (Fig 3). The posterolateral portal is located 2 cm proximal to the tip of the olecranon, adjacent to the lateral border of the triceps tendon. The trocard is directed toward the olecranon fossa, passing through the triceps to reach the posterior joint capsule. The posterior portal is midway between the epicondyles, between 2 and 4 cm from the tip of the olecranon to the middle of the triceps. The arthroscope was introduced through the posterolateral approach after filling the elbow with isotonic saline serum to move the vascular-nervous elements away from the bone. The second entry point was located at the lateral edge of the triceps tendon to introduce other instruments (Fig 3). The olecranon fossa was hollowed out with radiofrequency energy until bone contact, avoiding damage to the cartilage border of the trochlea. The osteochondromatous fragments were removed with a surgical clamp (Fig 4). A reaming was required to release the ossified areas, the osteophytes, and the olecranon bone spur (Fig 5). The perforation and fenestration of the bottom of the olecranon fossa, respecting the pillars of the metaphysis of the distal humerus, used a 5.5-mm oval bur until the olecranon fossa was in communication with the coronoid fossa. The diameter of the humeral fenestration averaged 10 mm, allowing us to have adequate visualization to perform our anterior capsulectomy. The communication between the posterior and anterior compartments allowed resection of the dense fibrous tissues of the coronoid fossa and the extraction of foreign bodies with forceps from the anterior compartment (Fig 6). The anterior capsulectomy used radiofrequency energy (Fig 7) up to the brachial muscle fibers in front, followed by anterior synovectomy around the bone window and then extended throughout the entire arthroscopic work area. In some cases, resection of the coronoid process bone spurs was also performed through humeral fenestration under direct visual control. During each step of the intervention, the gain in mobility was tested. The surgery ended when the gain in mobility seemed maximal. At this time, abundant irrigation was required, followed by closure of incisions with interrupted sutures.

Operation duration and tourniquet duration were measured parameters. There were no postoperative posture adjustments. Mobilization of the elbow was carried out progressively from passive to active without limitation during the stay in the postoperative monitoring room. Physiotherapy was initiated as soon as the patient was discharged from the hospital.

### Evaluation

All functional data were collected by the treating surgeon during preoperative consultation, intraoperatively, and during the follow-up consultations at 6 weeks and at the longest follow-up:

• Elbow joint range of motion in flexion/extension and pronation/supination, expressed as gain and secondary loss of gain. Gain was defined as the difference between values of joint range of motion before the surgery and at each follow-up time. We then measured the loss of this clinical gain during follow-up. A goniometer was used. Range of motion was the primary outcome measure.
• The Mayo Elbow Performance Score was calculated preoperatively and at the longest follow-up.
• The occurrence of complications.

Patient follow-up ended when the elbow became asymptomatic again. All elbows underwent anteroposterior and lateral x-rays during the postoperative follow-up consultations.

### Statistical Analysis

Statistical analysis was performed by a statistician on each patient’s data (joint range of motion and the Mayo Elbow Performance Score). After performing an analysis of variance, 3 multiple comparison tests for all pairwise differences between the means were compared: Bonferroni multiple comparison test, Fisher least significant difference multiple comparison test, and Tukey-Kramer multiple comparison test. Among the 3, the most precise test was the Tukey-Kramer multiple comparison test, which was therefore chosen for the statistical analysis of the study. The minimum expected clinical difference in improved elbow mobility was a 20% gain in flexion/extension at the longest follow-up. The chosen threshold of statistical significance was *P* < .05.

## Results

Our study included 30 patients with a male predominance of 76.7% (23 men vs 7 women). One patient was excluded from the study because an olecranon osteosynthesis had to be removed by an open approach. A total of 31 elbows (14 left and 17 right; 29 unilateral and 1 bilateral) underwent arthroscopic arthrolysis using a posterior approach via Outerbridge-Kashiwagi humeral fenestration. The mean age was 41.8 years with an age range from 19.5 to 64.9 years. The average follow-up was 11.1 months (1-20.3). The causes of elbow stiffness were degenerative in 12 patients (38.7%), post-traumatic in 14 patients (45.2%), inflammatory in 2 patients (6.5%), and 3 hemophilic arthropathies (9.7%). Humeral fenestration and anterior capsulectomy were performed in 100% of cases. There were 6 open conversions (19.4%), 2 cases due to the extraction of oversized foreign bodies, and 4 cases due to excessively long tourniquet times. Two patients presented postoperative complications (6.4%): 1 early recurrent elbow stiffness in a patient with rheumatoid arthritis and 1 hematoma in a patient with severe hemophilia. There were no vascular, nerve, or septic complications. No patients required neurolysis of the ulnar nerve. Follow-up radiographs showed persistent humeral fenestration with no bone complications.^28^^,^^29^ Elbow joint range of motion was measured preoperatively, intraoperatively, at 6 weeks, and at the longest follow-up (Table 1), establishing relative mean gains and deficits in flexion/extension range of motion (Table 2) and pronation/supination range of motion (Table 3). Mean joint amplitudes intraoperatively increased in all areas of mobility, including extension/flexion from 86° to 132.6° (*P* = .001) and pronation/supination from 163.9° to 179.7° (*P* = .025). At the longest follow-up, mean joint amplitude was increased from 86° to 118.9° (*P* = .002) in extension/flexion and from 136.9° to 173.9° (*P* = .022) in pronation/supination. The preoperative mean values were significantly different from the intraoperative and postoperative mean values (at 6 weeks and at the longest follow-up). Postoperative mean values were not significantly different from each other. There was a significant improvement in joint range in flexion/extension means intraoperatively, postoperatively at 6 weeks, and the longest follow-up compared with preoperative means. The results expressed in Table 3 are mean values in pronation and supination. Preoperative mean values were significantly different from intraoperative mean values only. Postoperative mean values were not significantly different from each other. There was a significant improvement in the intraoperative joint range means in pronation/supination compared with the preoperative means. The results expressed in Table 4 are mean values, for the different mobility sectors, of the intraoperative gain and loss of gain at the longest follow-up. In each mobility sector, the measured loss of the gain was low (7.1% in flexion, 7.2% in extension, 1.3% in pronation, 4.4% in supination). The minimum expected clinical difference in improved elbow mobility was a 20% gain in flexion/extension at the longest follow-up. Sixty-eight percent of patients gained at least 20° of flexion/extension mobility.

**Table 1:** Patient Values of Preoperative, Intraoperative, and Longest Follow-Up in Extension (EXT), Flexion (FLE), Pronation (PRO), and Supination (SUP)

Patient Number	Preoperative (°)				Intraoperative (°)				Longest Follow-Up (°)			
	EXT	FLE	SUP	PRO	EXT	FLE	SUP	PRO	EXT	FLE	SUP	PRO
1	20	130	45	160	0	140	0	180	0	140	20	160
2	35	115	20	160	0	140	0	180	10	120	0	180
3	30	115	0	180	20	120	0	180	45	120	0	180
4	20	140	0	180	0	140	0	180	15	140	0	180
5	20	120	0	180	0	140	0	180	0	130	0	180
6	20	140	0	180	0	140	0	180	5	130	0	170
7	60	120	20	160	20	130	0	180	45	120	0	180
8	30	120	0	180	0	140	0	180	30	140	0	160
9	30	110	30	140	10	120	0	170	30	100	20	120
10	60	120	0	180	40	140	0	180	40	115	0	180
11	40	120	0	180	10	140	0	180	10	140	0	180
12	20	140	0	180	0	140	0	180	5	135	0	180
13	40	140	0	180	0	140	0	180	5	140	0	180
14	30	120	0	180	0	140	0	180	20	130	0	180
15	10	120	0	160	0	140	0	180	10	120	0	180
16	80	120	0	180	10	140	0	180	5	120	0	180
17	5	110	0	160	10	140	0	180	10	130	0	160
18	20	115	0	180	0	140	0	180	0	140	0	180
19	15	100	40	160	0	140	0	180	15	120	0	180
20	15	105	0	180	5	130	0	180	0	135	0	180
21	45	120	0	180	10	140	0	180	15	125	0	180
22	80	100	60	120	15	140	0	180	45	120	0	160
23	40	110	20	150	10	140	0	180	10	130	0	180
24	50	140	0	180	0	140	0	180	15	140	0	180
25	40	130	0	180	0	140	0	180	0	140	0	180
26	30	115	0	180	10	140	0	180	10	140	0	180
27	30	140	0	180	0	140	0	180	0	140	0	180
28	30	120	0	180	0	140	0	180	0	140	0	180
29	40	120	0	180	0	140	0	180	0	140	0	180
30	40	100	0	180	0	120	0	180	0	130	0	180
31	40	115	0	180	0	140	0	180	0	130	0	180

**Table 2:** Mean Values of Joint Range of Flexion (F) and Extension (E)

Mean Values	Preoperative	Intraoperative	At 6 Weeks	Longest Follow-Up
Joint range of F/E (*P* < .05)	86°	132.6°	117.9°	118.9°
Deficit of F/E (*P* < .05)	54°	7.4°	22.1°	21.1°
Deficit of F (*P* < .05)	19.7°	2.6°	10°	9.7°
Deficit of E (*P* < .05)	34.4°	5.5°	12.7°	12.7°
Gain of F/E (*P* < .05)		46°	31.3°	31.5°
Gain of F (*P* < .05)		17.1°	9.7°	10°
Gain of E (*P* < .05)		28.9°	21.6°	21.6°

**Table 3:** Mean Values of Joint Range of Pronation (P) and Supination (S)

Mean Values	Preoperative	Intraoperative	At 6 Weeks	Longest Follow-Up
Joint range of P/S (*P* < .05)	163.9°	179.7°	177.4°	173.9°
Deficit of P/S (*P* < .05)	16.1°	0.3°	2.6°	6.1°
Deficit of P (*P* < .05)	8.4°	0.3°	2.6°	4.8°
Deficit of S (*P* < .05)	7.9°	0°	0°	1.3°
Gain of P/S (*P* < .05)		15.8°	13.5°	10°
Gain of P (*P* < .05)		7.7°	5.4°	3.2
Gain of S (*P* < .05)		7.6°	7.6°	6.3°

**Table 4:** Mean Values of Intraoperative Gain and Loss of Gain at the Longest Follow-Up in Flexion, Extension, Pronation, and Supination

Mean Values	Preoperative gain	Loss of Gain at Longest Follow-Up
Flexion	17.1°	7.1°
Extension	28.9°	7.2°
Pronation	7.8°	1.3°
Supination	7.6°	4.4°

The Mayo Elbow Performance Score increased between the preoperative consultation and the longest follow-up from 77.2 to 98.5 of 100 points (*P* = .003). Subscore analysis revealed a significant improvement: 26.6 of 45 intraoperatively versus 44 of 45 points at the longest follow-up (*P* = .012) for pain and 16.8 intraoperatively versus 19.5 of 20 points at the longest follow-up (*P* = .02) for mobility. The differences in the other subscores (stability and activity) were statistically insignificant.

**Fig 8:**
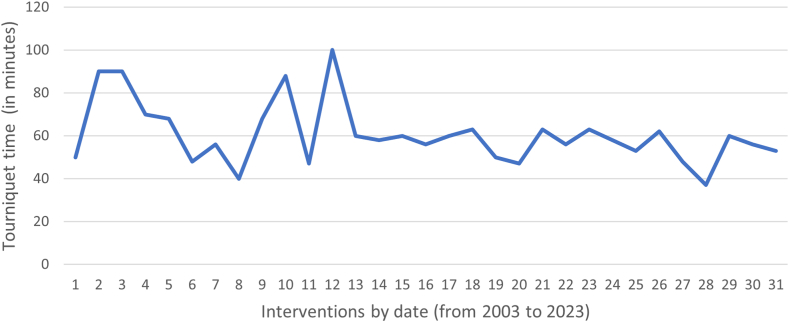
Evolution of tourniquet time during the surgery. The graphic shows the evolution of the tourniquet in relation to successive procedures from 2003 to 2023. There was an initial period of around 15 arthroscopic arthrolysis procedures, with variability in operation time, followed by a tendency for tourniquet time to decrease and stabilize.

Figure 8 shows the evolution of tourniquet time in minutes in relation to successive procedures from 2003 to 2023. The mean tourniquet duration was 61 minutes (37-100) over the 31 surgical operations, with 6 operations requiring conversion (90 minutes, 90 minutes, 90 minutes, 70 minutes, 90 minutes, 58 minutes). There was an initial period of around 15 arthroscopic arthrolysis procedures, with variability in operation time, followed by a tendency for tourniquet time to decrease and stabilize.

## Discussion

This study of 31 elbows showed that arthroscopic arthrolysis using humeral fenestration showed a significant improvement in elbow joint arc of motion without nerve complications. This technique was successful in the current practice without an anterolateral approach.

The gain in joint amplitude and the complication rates observed were comparable with data in the literature on conventional arthrolysis using an anterior approach.^3^^,^^25^^,^^30^^,^^31^

The Outerbridge-Kashiwagi humeral fenestration technique enabled anterior capsulectomy to be performed via a posterior approach, with mobilization and extraction of anterior foreign bodies through the humeral window^24^ and the possibility of resecting anterior osteophytes during fenestration.^32^ Anterior capsulectomy was an indispensable procedure in the treatment of elbow stiffness, as it increased release in both extension and flexion.^9^^,^^31^

The supination/pronation arc of mobility was only slightly deficient preoperatively but not negligible. The overall gain was of the order of 15°. There was a tendency to improve supination rather than pronation, as previously reported.^30^

Indications according to the cause of stiffness were similar to those in the literature.^9^ In our sample of 31 elbows, the causes were mainly post-traumatic (45.2%) and degenerative (38.7%). Inflammatory causes are at greater risk of secondary restiffening.^33^ In our series, we had a patient with rheumatoid arthritis who had recurrent stiffness. She underwent total arthroplasty at the age of 33 years. The study included 3 patients with hemophilic arthropathy, one of whom developed a hematoma complication. The occurrence of hemarthroses was reduced by surgery,^34^ with extensive synovectomy.

In the same way as the reeducation protocol described by Morrey^32^ in 1990, we recommended a passive and active progressive rehabilitation immediately to prevent the negative effects of immobilization, with additional physiotherapy and the introduction of diclofenac for 5 days to prevent joint capsule ossifications. More “aggressive” rehabilitation did not improve long-term results, as shown in the literature.^32^ We did not use continuous passive motion with an articulated splint recommended by Morrey.^32^

Based on the results obtained, significant restiffening was observed in all sectors at 6 weeks compared with the amplitudes measured at the end of the operation. Tissue healing takes around 6 weeks, by which time the patient is at a distance from surgical complications. The results between the 6-week follow-up visit and the longest follow-up showed a progression of gains in all sectors.

Arthroscopic elbow arthrolysis is a complex procedure requiring a certain mastery of arthroscopy. The learning curve for this complex procedure is poorly described in the literature. Kim et al.^35^ studied the learning curve for conventional arthroscopic release using an anterior approach by several surgeons. Their criterion was the tourniquet time required per procedure, and they observed the evolution of this time as a function of the surgeon’s experience (determined by the number of anterior elbow arthrolysis surgeries performed). The tourniquet time required for the procedure seemed to stabilize from the 15th experience onward and averaged 104 minutes. In our study of arthroscopic elbow arthrolysis using posterior humeral fenestration, we looked at the same criterion, recording the tourniquet time required for each procedure. The evolution of tourniquet time in the study also showed improvement and stabilization of the interventional duration from the 15th procedure, with a lower mean tourniquet time, measured at 61 minutes. This difference can be explained by the improvements in arthroscopy equipment.

### Limitations

This study is not without limitations. First, it is retrospective, which impacts the quality and availability of data. The patient sample size was small, so we cannot rule out the possibility for a type II error. In addition, the average follow-up was short, only 11 months. The study is monocentric; the spectrum of patients is therefore reduced, with less applicability of the results obtained to the general population than if the study had been multicentric. There is no comparative analysis directly to a cohort of patients treated with open releases. Patients were operated on and followed up by the same operator. This reduces the factors of variability in surgical technique and consultation evaluation criteria, and it allows us to observe the surgeon’s learning curve. But a single-operator study also reduces the applicability of the results to the general population. We included only 30 patients over a 20-year span; therefore, this study is limited in actually discerning the learning curve and improvement in tourniquet time over the 20-year period.

## Conclusions

Arthroscopic arthrolysis of a stiff elbow using a purely posterior approach with anterior capsulectomy via the Outerbridge-Kashiwagi procedure was safe and effective. Clinical results showed improvement in joint range of motion in flexion/extension and pronation/supination, both intraoperatively and postoperatively, with no postoperative neurologic complications.

## Disclosures

All authors (C.L., A.S., F.D.) declare that they have no known competing financial interests or personal relationships that could have appeared to influence the work reported in this paper.
